# 

**Published:** 1876-01

**Authors:** 


### ANNOUNCEMENTS FOR THE MONTH.

### MONDAYS.

SOCIETIES.

*Mondays, Jan.* 3 and 17—Chicago Med. Society, regular meetings at Gault House, 8 P.M.

*Mondays, Jan.* 10 and 24 — Chicago Society of Physicians and Surgeons, regular meetings at Grand Pacific, 8 P. M.

CLINICS. *Every Monday.*

At Eye and Ear Infirmary, (Peoria and Adams Sts.) 2 P. M.—Prof. Holmes.

At Chicago College, 2 P. M. *Gynecological*—Prof. Merriman.

At Central Dispensary (239 W. Van Buren St.), 2 P.M., *Gynecological*—Dr. Adolphus.

At Mercy Hospital, 2 P. M. *Medical*—Prof. Johnson.

LECTURES. *Every Monday.* At Rush College (18th and Arnold Sts.), 8½ to 12½ o'clock—Profs. Gunn, Miller, Freer and Powell; 4 to 6—Profs. Lyman and Etheridge. At Chicago College, 8½ to 12½—Profs. Jewell, Isham, Hyde and Bond; 3 to 6—Profs. Davis and Nelson, Andrews and Quine, and Roler. At Womans' Hospital College, 9 to 12—Profs. Thompson, Bogue and Blake; 3 to 6—Profs. Paoli, Stevenson and MacDonald.

### TUESDAYS.

SOCIETIES.

*Tuesday, Jan.* 11— Academy of Sciences, annual meeting, 8 P. M. (263 Wabash Av).

*Tuesday, Jan.* 25—Medico-Historical Society, regular meeting, 8 P. M.

CLINICS. *Every Tuesday.*

At County Hospital, 2 P. M., *Medical*—Prof. Ross; 3 P. M., *Surgical*—Prof. Bogue.

At Chicago College, 2 P. M., *Gynecological*—Prof. Roler.

At Mercy Hospital, 2 P. M., *Medical*—Prof. Hollister.

LECTURES. *Every Tuesday.*

At Rush College, 8½ to 12½—Profs. Gunn, Miller, Allen and Parkes; 4—Prof. Lyman. At Chicago College, 8½ to 12½—Profs. Jewell, Isham, Merriman and Bond; 3 to 6—Profs. Davis and Nelson, Andrews and Hatfield, and Byford. At Woman's Hosp. Coll., 9 to 12—Profs. Marguerat, Bartlett and Fitch;—3 to 6, Profs. Delafontaine, Curtis and MacDonald.

### WEDNESDAYS.

CLINICS. *Every Wednesday.*

At County Hospital, 2 P. M., *Ophthalmological*—Dr. Montgomery; 3 P. M., *Gynecological* —Prof. Fitch.

At Chicago College, 2 P. M., *Gynecological*—Prof. Nelson.

At Mercy Hospital, 2 P. M., *Surgical*—Prof. Andrews.

LECTURES. *Every Wednesday.*

At Rush College, 8½ to 12½—Profs. Hay, Freer, Allen and Parkes; 4 to 6—Profs. Lyman and Etheridge. At Chicago College, 8½ to 12½—Profs. Hollister, Isham, Hyde and Bond; 3 to 6—Profs. Davis and Curtis, Jones and Quine, and Roler. At Woman's Hosp. Coll., 9 to 12—Profs. Dyas, Earle and Thompson; 3 to 5—Profs. Paoli and Stevenson.

### THURSDAYS.

CLINICS. *Every Thursday.*

At Eye and Ear Infirmary, 2 P. M.—Prof. Holmes.

At Chicago College, 2 P. M., *Gynecological*—Prof. Merriman.

At Central Dispensary, 2 P. M., *Gynecological*—Dr. Adolphus.

At Mercy Hospital, 2 P. M., *Medical*—Prof. Johnson.

LECTURES. *Every Thursday.*

At Rush College, 8½ to 12½—Profs. Gunn, Miller, Allen and Parkes; 4 to 6—Profs. Ross and Lyman. At Chicago College, 8½ to 12½—Profs. Hollister, Isham, Merriman and Bond; 3 to 6—Profs. Davis and Nelson, Andrews and Hatfield, and Byford. At Woman's Hosp. Coll., 9 to 12—Profs. Marguerat, Bogue and Fitch; 3 to 6—Profs. Delafontaine, Curtis and MacDonald.

### FRIDAYS.

SOCIETIES.

*Friday, Jan.* 14—State Microscopical Society of Illinois, regular meeting at the Academy of Sciences, 8 P. M.

CLINICS. *Every Friday.*

At County Hospital, 2 P. M., *Medical*—Prof. Ross; 3 P. M., *Surgical*—Prof. Bogue.

At Chicago College, 2 P. M., *Gynecological*—Prof. Roler.

At Mercy Hospital, 2 P. M., *On Dis. Eye and Ear*—Prof. Jones.

LECTURES. *Every Friday.*

At Rush College, 8½ to 12½—Profs. Gunn, Freer, Allen and Parkes; 4 to 6—Profs. Holmes and Etheridge. At Chicago College, 8½ to 12½—Profs. Hollister, Quine, Hyde and Bond; 3 to 6—Profs. Jones and Curtis, Andrews and Quine, and Roler. At Woman's Hosp. Coll., 9 to 12—Profs. Hotz, Bartlett and Blake; 3 to 5—Profs. Paoli and Stevenson.

### SATURDAYS.

CLINICS. *Every Saturday.*

At Rush College, 2 P. M., *Surgical*—Prof. Gunn; 3 P. M., *Diseases of the Brain and Nervous System*—Dr. Hay.

At Chicago College, 2 P. M., *Gynecological*—Prof. Nelson; *Surgical*—Prof. Andrews; 3 P. M., *Medical*—Prof. Davis.

LECTURES. *Every Saturday.*

At Rush College, 8½ to 12½—Profs. Hay, Miller, Freer and Parkes. At Chicago College, 8½ to 12½—Profs. Hollister, Quine, Sherman and Hatfield; 3 to 6—Profs. Davis and Nelson, Andrews and Quine, and Byford. At Woman's Hosp. College, 9 to 12—Profs. Marguerat, Earle and Fitch; 4 to 6—Profs. Curtis and MacDonald; 3 to 5—Prof. Thompson.

☞ There are six special Clinics daily at the South Side Dispensary (at Chicago College) for students of the Chicago College. A daily Clinic is given at Dispensary of Hospital for Women and Children, 1½ P. M., for students of Woman's Hospital College.

